# Familial neuralgia of occipital and intermedius nerves in a Chinese family

**DOI:** 10.1007/s10194-011-0350-4

**Published:** 2011-05-24

**Authors:** Yu Wang, Chuan-Yong Yu, Lin Huang, Franz Riederer, Dominik Ettlin

**Affiliations:** 1 Department of Neurology, Epilepsy and Headache Group, Institute of Neurology, The First Hospital of Anhui Medical University, Anhui Medical University, Jixi Road 218, Hefei, 230022 China; 2 Department of Neurology, University Hospital Zurich, Plattenstrasse 11, 8032 Zurich, Switzerland

**Keywords:** Occipital neuralgia, Nervus intermedius neuralgia, Familial syndrome, Inheritance patterns, Autosomal dominant inheritance, X-linked dominant inheritance

## Abstract

Cranial nerve neuralgia usually occurs sporadically. Nonetheless, familial cases of trigeminal neuralgia are not uncommon with a reported incidence of 1–2%, suggestive of an autosomal dominant inheritance. In contrast, familial occipital neuralgia is rarely reported with only one report in the literature. We present a Chinese family with five cases of occipital and nervus intermedius neuralgia alone or in combination in three generations. All persons afflicted with occipital neuralgia have suffered from paroxysmal ‘electric wave’-like pain for years. In the first generation, the father (index patient) was affected, in the second generation all his three daughters (with two sons spared) and in the third generation a daughter’s male offspring is affected. This familial pattern suggests an X-linked dominant or an autosomal dominant inheritance mode.

## Introduction

Occipital neuralgia (ON) refers to a paroxysmal stabbing pain, with or without persistent aching between paroxysms, in the distributions of the greater or lesser occipital nerves [11]. Nervus intermedius neuralgia (NIN) usually provokes a very intense and stabbing pain localized in the depth of the ear canal, which is an uncommon disorder that affects a sensory branch of the facial nerve [11]. These two cranial nerve neuralgias are uncommon and usually occur in isolated and sporadic fashion. Only one report described combined occurrence and familial clustering in a Swiss family which sparked our interest in reporting the present familial presentation [14]. With regard to familial clustering of cranial neuralgias, it is known that comparatively common trigeminal nerve neuralgia (TNG) may have a positive family history in 1–2% cases indicating a possible autosomal dominant (AD) inheritance [10, 16]. We present a Chinese family with five cases of occipital and nervus intermedius neuralgia alone or in combination in three generations, suggestive of an X-linked dominant (XLD) or an AD inheritance mode.

## Case report

An 89-year-old man presented with a history of episodes of a paroxysmal headache on the right occipital region, superior and posterior to the ear, over the past 40 years. He complained of abrupt onset, sharp, electric-like pain in the right occiput radiating toward the vertex accompanied by an intense and stabbing pain localized in the depth of the auditory canal. Palpation of the right greater occipital nerve elicited the pain. A single pain episode usually lasted for a few weeks. The pain in the depth of the right auditory canal did not appear during every episode but only when the occipital pain was severe. The pain attacks usually occurred at the time the patient felt fatigued or when he was afflicted by a cold with or without fever. During the latest episode, the occipital pain was accompanied by symptoms of a herpes zoster and/or herpes simplex reactivation in the territories of the right maxillary and auriculotemporal nerves. Specifically, the skin was erythematous and small vesicles appeared on the right side of the face, around the right mouth angle and the right external auditory canal. The viral smear was not obtained and the vesicles resolved spontaneously in weeks. The frequency of the pain attacks was increasing from once in every 1 or 2 years in the antecedent decade to two or three times per year in recent years. Neurological examination revealed normal findings with no hypesthesia in the affected area, no cranial autonomic features, and no occipital skin lesions. Pain was eased temporarily by local anesthetic nerve block with lidocaine. Magnetic resonance image (MRI) examination of the cervical spine and brain was normal. A diagnosis of ON in combination with NIN was established based on symptoms which fulfilled the International Headache Society (ICHD-II) criteria [11]. In the antecedent decade, the patient did not take any pain killer. In recent years, carbamazepine was used at a dosage of 0.3 g daily (divided into three times) during the pain attacks and the intensity and frequency of the pain attacks could be eased, but the episodic duration of pain attacks was not reduced.

## Family history

**Fig. 1:**
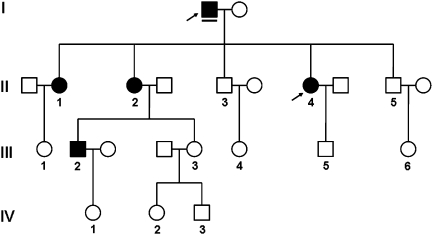
Pedigree of occipital and nervus intermedius neuralgia. *Squares* represent males; *cycles* females. *Filled symbols* indicate individuals with occipital neuralgia; *open symbols*, unaffected status; *symbols with arrows* point to individuals with neuralgia of nervus intermedius. The index patient is *underlined*. *I*–*IV* indicate the generations from the first to the fourth. Ages of the third and the fourth generations: *III*.*1* 32 years, *III*.*2* 37 years, *III*.*3* 35 years, *III*.*4* 30 years, *III*.*5* 26 years, *III*.*6* 26 years, *IV*.*1* 5 years, *IV*.*2* 8 years, *IV.3* 5 years

The index patient had a family history of ON which had been diagnosed in four other family members. He had three daughters and two sons. One of his daughters (third born, II. 4 in Fig. 1) has ON in combination with NIN, and the two others (II. 1 and 2 in Fig. 1) have ON only. The two sons (II. 3 and 5 in Fig. 1) are spared of ON and NIN, and none of their offsprings (III. 4 and 6 in Fig. 1) have ON or NIN. The index patient has six grandchildren: two grandsons and four granddaughters. One grandson (son of the second born daughter, III. 2 in Fig. 1) has ON alone without combined NIN, and the other grandson (III. 5 in Fig. 1) and all the granddaughters are spared of ON and NIN. In the third generation, the eldest person is the affected grandson currently aged 37. This family history is summarized in a pedigree figure (Fig. 1).

The two elder daughters (II. 1 and 2 in Fig. 1) of the index patient developed abrupt-onset, sharp, electric-like pain in the distribution of the occipital nerve at the age of 50 and 55, respectively. These two elder daughters never had ear canal pain prior to the onset of the electric-like pain in the distribution of the occipital nerve. The third daughter (II. 4 in Fig. 1) had the same symptoms like her father that began at the age of 43 with electric-like pain in the distribution of the occipital nerve and pain in the depth of the ear canal. The three daughters had these pain symptoms for 12, 6 and 11 years, respectively with the same pain attack frequency: one episodic attack every 1 or 2 years. The pain attacks occurred predominantly at the time they felt fatigued and at the time they were afflicted by a cold with or without fever. The second daughter also had an intercostal neuralgia caused by herpes zoster recently at the age of 62. The medical and neurological examinations were normal for all of them. The second daughter also had an MRI examination with the result of normal cervical spine and brain image. Based on the International Headache Society (ICHD-II) criteria [11], a diagnosis of ON and/or NIN was established. All women had an optimal response to carbamazepine treatment during the pain attack, which was similar to their father’s.

The grandson (III. 2 in Fig. 1), son of the index patient’s second daughter was first affected at the age of 33. He had two episodes of abrupt-onset, sharp, electric-like pain in the distribution of the occipital nerve in the past 4 years. The pain attacks occurred one time when he was very busy with limited sleep time, another time after heavy alcohol consumption. He also had an optimal response to carbamazepine treatment during the pain attack, which was similar to his grandfather’s.

## Discussion

The International Headache Society (ICHD-II) criteria [11] for ON includes: (1) paroxysmal stabbing pain, with or without persistent aching between paroxysms, in the distribution(s) of the greater, lesser and/or third occipital nerves; (2) tenderness over the affected nerve; and (3) pain eased temporarily by local anaesthetic block of the nerve. Our index patient and his third daughter fully fulfilled the current IHS criteria for ON, and two other family members fulfilled the current IHS criteria for ON except one criterion; namely, a decrease in pain by anesthetic block. Most ONs are idiopathic, but in rare cases, they may be a result of cervical spine osteochondroma [1], cavernoma [4, 5], multiple sclerosis, schwannoma [2, 8], meningioma [12], and myelitis [3, 9]. Though MRI examination was not conducted in all family members, the MRI results of the index patient and his second daughter had excluded local pathologies thus indicating idiopathic ON. Our patient and his third daughter also had combined auditory canal pain with the presence of a trigger area in the posterior wall of the auditory canal which fitted the ICHD-II criteria for NIN, thus a diagnosis of ON in combination with NIN was established in these two cases of this family.

Microvascular compression is an accepted cause of classic TNG but little is known about the etiology of idiopathic ON and NIN, apart from two articles suggestive of microvascular compression. One article about the anatomy of occipital nerve in a postmortem study showed that occipital artery and occipital nerve crossed each other and an occipital nerve has indentation by the occipital artery at the crossing point in all specimens studied [15]. This may be suggestive of microvascular compression in ON, though direct evidence is lacking. A case report showed vascular compression of the nervus intermedius by the anterior inferior cerebellar artery, and the patient’s pain was successfully managed with microvascular decompression [17]. Thus, ON and NIN may share the same pathology of microvascular compression with TGN. And immunological abnormalities or infection may function as triggers of this neuralgia, because the neuralgia usually recurred while they were feeling fatigued or had a common cold. This is in consistence with a recent report showing that ON was secondary to respiratory tract infection [13]. Our patient and his second daughter also had single episodes of herpes zoster.

The most important finding in our cases is that the neuralgia occurred in every generation of a family. This is the second report of familial ONs with only one previous report of a Swiss family in which a man was affected in the first generation and all women but none of the men were affected in the second generation [14]. In the current report, the same transmission mode was observed but across three generations. Our cases combined with the recently reported familial cases suggest that familial ON may be characterized by XLD or AD transmission. The observation that affected individuals in the current family had optimal treatment responses to the sodium channel blocker carbamazepine leads to the speculation that familial neuralgia may be linked to a sodium channel mutation. These channels play a major role in the pathogenesis of neuropathic pain. For example, the gain-of-function mutation of one particular sodium channel, Nav1.7, causes inherited erythromelalgia, an AD inherited painful neuropathy, and paroxysmal extreme pain disorder [7]. This Nav1.7 mutation results in relatively large responses to small, subthreshold depolarizations, i.e. hyperexcitability of DRG neurons [6]. Thus, we hypothesize that there may exist familial inherited hyperexcitability of DRG neurons and that these neurons may be easily responsive to immunological imbalances.

Further reports of similar cases may help to improve our understanding of the nature of idiopathic neuralgias and to develop optimized therapeutic management.

## References

[1] Baer-Henney S, Tatagiba M, Samii M (2001). Osteochondroma of the cervical spine causing occipital nerve neuralgia. Case report. Neurol Res.

[2] Ballesteros-Del Rio B, Ares-Luque A, Tejada-Garcia J, Muela-Molinero A (2003). Occipital (Arnold) neuralgia secondary to greater occipital nerve schwannoma. Headache.

[3] Boes CJ (2005). C2 myelitis presenting with neuralgiform occipital pain. Neurology.

[4] Bruti G, Mostardini C, Pierallini A, Villani V, Modini C, Cerbo R (2007). Neurovascular headache and occipital neuralgia secondary to bleeding of bulbocervical cavernoma. Cephalalgia.

[5] Cerrato P, Bergui M, Imperiale D, Baima C, Grasso M, Giraudo M, Lentini A, Lopiano L, Bradac GB, Bergamasco B (2002). Occipital neuralgia as isolated symptom of an upper cervical cavernous angioma. J Neurol.

[6] Cummins TR, Dib-Hajj SD, Waxman SG (2004). Electrophysiological properties of mutant Nav1.7 sodium channels in a painful inherited neuropathy. J Neurosci.

[7] Dib-Hajj SD, Black JA, Waxman SG (2009). Voltage-gated sodium channels: therapeutic targets for pain. Pain Med (Malden, Mass).

[8] Garza I (2007). Craniocervical junction schwannoma mimicking occipital neuralgia. Headache.

[9] Goicochea MT, Romero C, Leston JA (2008). Occipital neuralgia with cervical myelitis. Cephalalgia.

[10] Harris W (1940). An analysis of 1,433 cases of paroxysmal trigeminal neuralgia and the end results of gasserian alcohol injection. Brain.

[11] IHS (2010) Classification ICHD-II. Cranial neuralgias and central causes of facial pain http://ihs-classification.org/en/02_klassifikation/04_teil3/13.00.00_facialpain.html (viewed 3 May 2011)

[12] Kim NH, Yang SY, Koo JB, Jeong SW (2009). Occipital neuralgia as the only presenting symptom of foramen magnum meningioma. J Clin Neurol (Seoul, Korea).

[13] Mourouzis C, Saranteas T, Rallis G, Anagnostopoulou S, Tesseromatis C (2005). Occipital neuralgia secondary to respiratory tract infection. J Orofac Pain.

[14] Riederer F, Sandor PS, Linnebank M, Ettlin DA (2010). Familial occipital and nervus intermedius neuralgia in a Swiss family. J Headache Pain.

[15] Shimizu S, Oka H, Osawa S, Fukushima Y, Utsuki S, Tanaka R, Fujii K (2007). Can proximity of the occipital artery to the greater occipital nerve act as a cause of idiopathic greater occipital neuralgia? An anatomical and histological evaluation of the artery-nerve relationship. Plast Reconstr Surg.

[16] Smyth P, Greenough G, Stommel E (2003). Familial trigeminal neuralgia: case reports and review of the literature. Headache.

[17] Younes WM, Capelle HH, Krauss JK (2010). Microvascular decompression of the anterior inferior cerebellar artery for intermediate nerve neuralgia. Stereotact Funct Neurosurg.

